# Informatics-Based Discovery of Disease-Associated Immune Profiles

**DOI:** 10.1371/journal.pone.0163305

**Published:** 2016-09-26

**Authors:** Amber Delmas, Angelos Oikonomopoulos, Precious N. Lacey, Mohammad Fallahi, Daniel W. Hommes, Mark S. Sundrud

**Affiliations:** 1 Department of Cancer Biology, The Scripps Research Institute, Jupiter, Florida, United States of America; 2 Department of Immunology and Microbial Sciences, The Scripps Research Institute, Jupiter, Florida, United States of America; 3 Division of Digestive Disease, University of California Los Angeles, Los Angeles, California, United States of America; 4 Informatics Core, The Scripps Institute, Jupiter, Florida, United States of America; Karolinska Institutet, SWEDEN

## Abstract

Advances in flow and mass cytometry are enabling ultra-high resolution immune profiling in mice and humans on an unprecedented scale. However, the resulting high-content datasets challenge traditional views of cytometry data, which are both limited in scope and biased by pre-existing hypotheses. Computational solutions are now emerging (e.g., Citrus, AutoGate, SPADE) that automate cell gating or enable visualization of relative subset abundance within healthy versus diseased mice or humans. Yet these tools require significant computational fluency and fail to show quantitative relationships between discrete immune phenotypes and continuous disease variables. Here we describe a simple informatics platform that uses hierarchical clustering and nearest neighbor algorithms to associate manually gated immune phenotypes with clinical or pre-clinical disease endpoints of interest in a rapid and unbiased manner. Using this approach, we identify discrete immune profiles that correspond with either weight loss or histologic colitis in a T cell transfer model of inflammatory bowel disease (IBD), and show distinct nodes of immune dysregulation in the IBDs, Crohn’s disease and ulcerative colitis. This streamlined informatics approach for cytometry data analysis leverages publicly available software, can be applied to manually or computationally gated cytometry data, is suitable for any clinical or pre-clinical setting, and embraces ultra-high content flow and mass cytometry as a discovery engine.

## Introduction

In the current era of high content flow cytometry (FACS), and even higher content mass cytometry (CyTOF), large-scale immune profiling in mice and human patients is becoming commonplace [1–3]. However, the value of using ultra-high content cytometry as a discovery tool is undermined by traditional views of FACS data, and current methods of FACS analysis. Even a single 10-parameter FACS experiment performed on human PBMC or mouse splenocytes can distinguish–assuming simple bi-modal distribution–up to 1,024 (2^10^) distinct immune phenotypes. Yet only a fraction of this information is used because it is not practical to analyze hundreds of individual immune phenotypes “one-by-one”. Rather, cytometry data is routinely distilled down to focus on small numbers of well-characterized immune cell subsets that fit a hypothesis; this in turn generates bias, and disregards a large amount of potentially valuable information.

Computational solutions (e.g., Citrus, AutoGate) have been developed to remove bias introduced by manual cell gating and identify phenotypically unique cell clusters [4,5]. Yet whether raw cytometry data is parsed manually or computationally, the challenge remains to link discrete immune phenotypes to disease endpoints of interest, both within and between groups of patients or experimental animals. Programs such as spanning tree progression of density-normalized events (SPADE) begin to address this issue [6]. Currently, however, SPADE is used largely as a visualization tool to display immunophenotypic differences between groups of healthy controls and patients [3,7,8], which ignores both within-group variability and immunophenotypic associations with clinical endpoints other than diagnosis. Thus, additional approaches are called for that enable a more personalized and dynamic view of immune profiling data.

Hierarchical clustering is a now-routine method of analyzing other forms of big data (e.g., transcriptomic profiling, genome sequencing), most commonly to identify groups of differentially expressed transcripts between distinct cell types, or between similar cell types isolated from different hosts [9,10]. Importantly, hierarchical clustering also identifies relationships (i.e., direct, inverse) between individual transcripts across all cells (i.e., within and between groups), which can then be used to infer gene regulatory networks [11]. However, the most common visual output of hierarchical clustering, the heatmap, lacks quantitative information about the similarity between variables. For this reason, many hierarchical clustering software packages, such as GenePattern (https://genepattern.broadinstitute.org), include nearest neighbor search algorithms (e.g., Euclidian distance, Manhattan distance, Pearson coefficient) that yield quantitative measurements of the similarity between variables [12]. Based on these concepts, we reasoned that combining hierarchical clustering with nearest neighbor searches in the context of immune profiling could identify relationships between large numbers of immune phenotypes and continuous clinical or pre-clinical disease endpoints. This “immuno-informatics” platform values increased readouts generated in ultra-high content cytometry experiments, can be applied downstream of manual or computational raw data analyses, and provides a comprehensive view of all immune phenotypes relative to discrete disease variables.

To explore the utility of this approach, we used manually gated immune profiling datasets from both a prevalent T cell transfer mouse model of chronic colitis [13], and human inflammatory bowel disease (IBD) patients. The T cell transfer model of colitis is interesting because the primary endpoints of disease are weight loss and histologic colitis, yet weight loss in this model is highly variable and does not necessarily correlate with severity of colitis [13]. Further, immune phenotypes that correlate with either weight loss or histologic colitis in this model are poorly characterized. By combining hierarchical clustering with nearest neighbor searches, we rapidly identify quantitative associations between specific immune phenotypes and disease endpoints, both in the T cell transfer model of colitis and human IBD patients.

## Materials and Methods

### Mice

Wild type FVB/N (FVB; model no. FVB) mice were purchased from Taconic. FVB.*Rag1*^-/-^ mice were provided by Dr. Allan Bieber (Mayo Clinic, Rochester, MN). All mice used in this study were housed, bred, used in experiments, and sacrificed in accordance with a protocol (13–019) approved by the Institutional Animal Care and Use Committee of Scripps Florida.

### Human samples

All experiments using human blood were conducted in accordance with IRB protocols approved by institutional review boards at The Scripps Research Institute or UCLA. Blood was obtained at UCLA following informed written consent from healthy adults, Crohn’s disease patients, or ulcerative colitis patients; consenting patients provided clinical history and demographic data at time of phlebotomy. The UCLA institutional review board approved all procedures and forms used to obtain informed patient consent, and all documentation for consenting patients is stored on paper at UCLA. PBMC was isolated and cryopreserved in de-identified and barcoded vials following ficoll density centrifugation, and frozen vials were shipped to Scripps Florida for analyses. PBMC vials were thawed and stained immediately for FACS analysis (see below).

### T cell transfer-induced colitis

CD4^+^CD25^-^ T cells from spleens and peripheral lymph nodes of wild type female FVB mice were magnetically isolated using an EasySep T cell negative isolation kit (Stem Cell Technologies, Inc.). Enriched splenocytes were further FACS-sorted to obtain pure naïve T cells (CD3^+^CD4^+^CD25^-^CD62L^hi^CD44^lo^); 0.5 x 10^6^ cells were injected intraperitoneally (i.p.) into 6- to 8-week old syngeneic female FVB.*Rag1*^-/-^ mice. *Rag1*^-/-^ mice were weighed directly prior to T cell transfer to obtain baseline weights; mice were weighed twice weekly for the duration of the experiments, and euthanized if ≥ 20% baseline weight loss was reached.

### Histology

Colons (~ 1 cm proximal, distal sections) were cut from euthanized *Rag1*^-/-^ mice 5–8 weeks post-T cell transfer (depending on animal morbidity) and fixed in 10% neutral buffered formalin, embedded into paraffin blocks, cut for slides, and stained with hematoxylin and eosin (H&E). H&E-stained sections were analyzed and scored blindly by a veterinary pathologist.

### Cell isolation

Mouse single mononuclear cell suspensions were prepared from spleen, or mesenteric lymph nodes (MLN) following tissue disruption. For isolation of mononuclear cells from colon, whole colons (cecum to anus) were removed, flushed with PBS to remove the fecal contents, and opened longitudinally to expose the epithelium. Tissues were incubated for 30 min at room temperature in DMEM media (without phenol red; Life Technologies) plus 0.15% DTT (Sigma-Aldrich) to remove mucus. After washing with media, colons were incubated for 30 min at room temperature in media containing 1 mM EDTA (Amresco) to remove the epithelium. After washing again with media, lamina propria was digested in media containing 0.25 mg/mL liberase TL and 10 U/mL RNase-free DNaseI (both from Roche), with shaking in a bacterial incubator (Environ Shaker; Labline) for 15–25 min at 37°C. Single cell suspensions were passed through 70 μm nylon filters (BD) and mononuclear cells were isolated by 70/30% percoll gradient centrifugation (Sigma-Aldrich). Mononuclear cells were washed twice in DMEM (LifeTech), counted, and resuspended for FACS analysis.

### Flow Cytometry

FACS staining for surface antigens was performed as previously described [14]. Intracellular stains were performed following 4 hr. *ex vivo* stimulation with phorbol 12-myristate 13-acetate (PMA) and ionomycin in the presence of brefeldin A (all from Sigma-Aldrich). Stimulated cells were washed in PBS, stained for cell surface antigens in PBS for 20 min. at room temperature, fixed and permeabilized using a Foxp3 intracellular staining kit (eBioscience), and then stained with antibodies against transcription factors and cytokines. Anti-mouse antibodies used for FACS analysis were: Alexa700-CD45, brilliant violet (BV)650-CD3, BV711-CD4, BV605-CD25, Percp/Cy5.5-CD44, BV605-CD62L, APC-IFNγ, Percp/Cy5.5-IL-17A, PE-IL-22, PE/Cy7-IL-10, FITC-Ki-67 (all from Biolegend); PE/CF594-CD25 and PE/CF594-RORγt (from BD); and e450-Foxp3 (clone FJK-16s) (from eBioscience). Anti-human antibodies used for FACS analysis were: APC-CD3, PE-CD4, PE/Cy7-CD45RO, Percp/Cy5.5-CCR7 (from Biolegend); and PE/CF594-CD25 (from BD). For both mouse and human FACS analysis, viable cells were discriminated using an eFluor® 506 fixable viability dye (eBioscience). All FACS data was acquired on LSRII and analyzed using FlowJo software (TreeStar, Inc.; version 9.8.5). Raw FACS data was analyzed by manual gating (using strategies shown throughout) following compensation set in FlowJo using single color-stained control samples. Subset frequencies (i.e., percentage of parent gates) and in some cases, absolute cell numbers (calculated by multiplying total mononuclear cell numbers by subset frequencies) were exported to Microsoft Excel. Absolute cell numbers were obtained for only spleen and mesenteric lymph node-derived subsets; cell numbers for subsets from colon lamina propria were not recorded or used for analyses given variable cell recovery from enzymatically-digested intestinal tissues.

### Bioinformatics analysis

**Fig 1:**
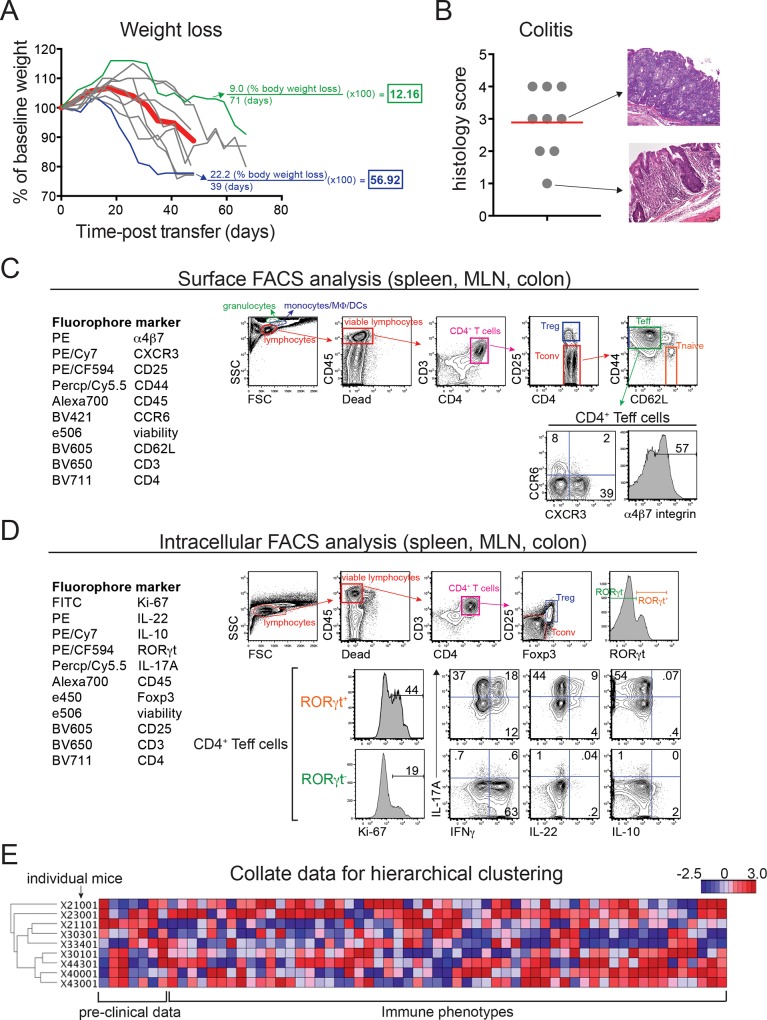
An informatics approach to correlating immune phenotpyes with weight loss or colitis in a T cell transfer mouse model of IBD. (A) Weight loss in FVB.*Rag1*^*-/-*^ mice (*n* = 9) injected with wild type naïve CD4^+^ T cells. Weights are shown relative to day 0 (pre-transfer baseline). Bold red trace shows mean weight loss for the group; green and blue traces show individual mice displaying mild or aggressive weight loss, respectively. Examples of disease severity index (DSI) calculations are shown in color-coded text. (B) Quantitative colitis scores (*n* = 9) from the same group of T cell-transferred FVB.*Rag1*^*-/-*^ mice shown in (A). H&E-stained colon tissues were scored blindly as in [17]; representative micrographs (at right) show mild (score of 1) and severe (score of 3) inflammation (20x magnification). Red horizontal bar indicates mean colitis scores for the group. (C) *Left*, 10-parameter FACS panel used for analyzing *ex vivo* expression of surface antigens on leukocytes isolated from spleen, mesenteric lymph nodes (MLN), and colon lamina propria (colon) of FVB.*Rag1*^*-/-*^ mice injected as in (A). *Right*, Gating strategy for surface FACS analysis; immune subsets used in downstream analysis are indicated by gates, text, and where appropriate, percentages. (D) *Left*, 11-parameter FACS panel used for analyzing *ex vivo* expression of intracellular transcription factors and cytokines in leukocytes isolated from T cell-transferred FVB.*Rag1*^*-/-*^ mice as above. *Right*, Gating strategy for intracellular FACS analysis; immune subsets used in downstream analysis are indicated by gates, text, and where appropriate, percentages. (E) Heat map showing hierarchical clustering of 7 disease endpoints and 57 immune phenotypes in T cell-transferred FVB.*Rag1*^*-/-*^ mice as above. Dendrograms (far left) show the clustering relationship between the mice based on all disease endpoints and immunophenotypes.

Immunophenotypic data from FlowJo as above were collated in Microsoft Excel together with clinical, pre-clinical, and human demographic data (converted to single numeric values; as in Fig 1A and 1B and Table 1). To enable analyses in GenePattern, Microsoft Excel spreadsheets containing the data were converted to gct files as per instructions found in the GenePattern File Formats Guide (http://software.broadinstitute.org/cancer/software/genepattern/file-formats-guide#GCT). gct files were then analyzed using the HierarchicalClustering module in GenePattern (http://genepattern.broadinstitute.org) using both row and column clustering (Pearson correlation) and log-transformation. Two-dimensional hierarchical clustering data output files (atr, cdt, gtr) where analyzed in the HierarchicalClusteringViewer module to generate heatmaps. Within the HierarchicalClusteringViewer software (run through a Java applet), “nearest neighbor searches” were performed to quantify similarity between select clinical or pre-clinical disease endpoints of interest and all other data. Pearson correlation was used for nearest neighbor searches unless noted otherwise. Euclidian or Manhattan distances were also tested in independent nearest neighbor searches to compare results with those generated using Pearson coefficients (S2 File). Follow-up analysis and graphing was performed using GraphPad Prism software.

**Table 1:** Conversion of IBD patient clinical and demographic data to single numeric values for hierarchical clustering.

Information	Values
Diagnosis group	healthy adults (1), UC (2), CD (3)
Age at diagnosis	as reported (no disease/healthy adults = 100*)
Age at collection	as reported
Gender	male (1), female (2)
Ethnicity	caucasian (1), african american (2), hispanic (3), asian (4), other minority (5)
No. of related surgeries	as reported (no surgeries = 0.01*)
Medications	none (1), 5-ASA (2), steroids (3), immunomodulator (4), biologic (5), combination of 2 (6), combination of 3 (7), combination of 4 (8)
Disease activity	inactive (1), minimal (2), mild (3), moderate (4), moderate/severe (5), severe (6)
History of ileitis	no (1), yes (2)

Qualitative clinical and demographic information was converted to single numeric values to enable hierarchical clustering with FACS-based immune profiling data. For each parameter, numbers in parentheses indicate the ascribed values used for hierarchical clustering. “As reported” indicates that no conversion was necessary beyond the information provided.

* In some cases (e.g., age at diagnosis in healthy controls), real numbers were created because GenePattern does not tolerate zero (0) values.

### Statistical analyses

Statistical analyses were performed in Prism (GraphPad). One-way ANOVA with no pairing and Tukey correction for multiple comparisons, as well as Pearson correlation tests were used as appropriate and are indicated in the Figure legends. For One-way ANOVA analyses, statistical comparisons were made between all groups; only significant differences (*P* ≤ .05) are shown in Figures.

## Results

### Distinct immune profiles correspond to T cell transfer-induced weight loss and colitis

To explore the utility of using informatics to link immune phenotypes with disease endpoints, we performed systematic immune profiling in a group of 9 *Rag1*^-/-^ mice transplanted with wild type naïve CD4^+^ T cells over 3 independent experiments. Transferred recipients were co-housed to normalize microflora, and both weight loss (Fig 1A), and histologic colitis was assessed (Fig 1B). At sacrifice, leukocytes from spleen, mesenteric lymph nodes (MLN), and colon lamina propria were analyzed by *ex vivo* surface (Fig 1C) or intracellular (Fig 1D) FACS to assess the phenotypes and absolute numbers of immune cell subsets, including: CD25^hi^Foxp3^+^ T regulatory (Treg) cells, CD25^lo^Foxp3^-^ T conventional (Tconv) cells, CD62L^lo^CD44^hi^ effector/memory T cells (Teff cells), and CD62L^hi^CD44^lo^ naïve T cells (Tnaive). Within Teff cells, we analyzed surface expression of pro-inflammatory chemokine receptors (e.g., CCR6, CXCR3), and the gut homing integrin **α**4**β**7 (Fig 1C). In addition, we analyzed intracellular expression of key transcription factors (Foxp3, ROR**γ**t), and–within Teff cells–expression of the proliferation-associated nuclear antigen, Ki-67 [15], as well as several pro- and anti-inflammatory cytokines (IL-17A, IFN**γ**, IL-22, IL-10) involved in mucosal immune regulation (Fig 1D) [16].

To enable hierarchical clustering of these immune phenotypes (57 in total) with weight loss or colitis, we converted information reflecting disease endpoints into single numeric values for each mouse. For weight loss, we created a disease severity index (DSI), which considers both the total percentage of bodyweight lost (relative to pre-transfer baseline) and the time in which weight loss occurs; higher values in this index reflect more aggressive weight loss (Fig 1A). For colitis, histologic inflammation was quantified using a standard scoring system of 0–4, where 0 reflects no evidence of inflammation and 4 indicates maximal severity of inflammation with transmural leukocyte infiltration and loss of goblet cells (Fig 1B) [17]. Other disease endpoints documented included colon weight (in g), colon length (in cm), and colon weight:length ratio, which correlate with histologic inflammation in some chemically induced models of colitis (data not shown) [17]. All data points (63 in total) were then collated into a single file for hierarchical clustering in GenePattern (http://genepattern.broadinstitute.org) (Fig 1E).

**Fig 2:**
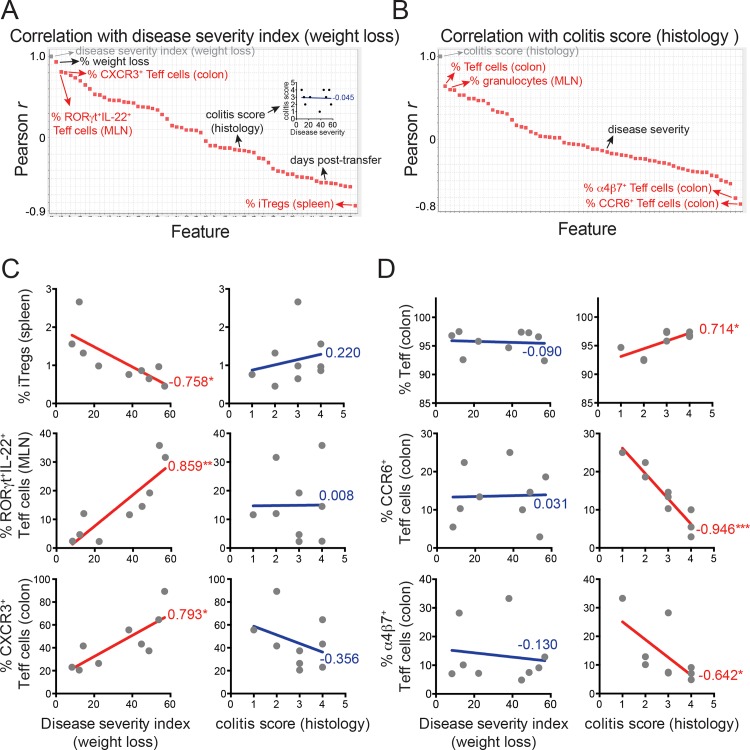
Discrete immune phenotypes correspond with T cell transfer-induced weight loss or colitis in *Rag1*^-/-^ mice. (A) Rank-ordered (Pearson *r*) correlation values of all disease endpoints and immune phenotypes relative to weight loss (disease severity index (DSI)), in FVB.*Rag1*^-/-^ mice injected with wild type naïve CD4^+^ T cells as in Fig 1A. Relevant disease endpoints and immune phenotypes are indicated by black and red text, respectively. Correlation between weight loss and colitis scores is further shown in insert, where blue text indicates the Pearson *r* correlation value. (B) Rank-ordered (Pearson *r*) correlation values of all disease endpoints and immune phenotypes relative to colitis scores, determined by histology, in the same T cell-transferred FVB.*Rag1*^-/-^ mice. Relevant immune phenotypes are indicated by red text; correlation with weight loss (DSI) is indicated by black text. For (A, B), the correlation of the reference variable with itself (*r* = 1.0) is shown at top left in grey. (C) Exemplar immune phenotypes that correlate with T cell transfer-induced weight loss (disease severity index (DSI)), (*left*), but not histologic colitis (*right*) in T cell-transferred FVB.*Rag1*^-/-^ mice. (D) Exemplar immune phenotypes that correlate with T cell transfer-induced colitis (*right*), but not weight loss (disease severity index (DSI)) (*left*). Pearson *r* correlation values are show in red (for correlations achieving statistical significance) and blue (for correlations not statistically significant). * P < .05, ** P < .01, *** P < .001, Pearson correlation test.

After clustering, we highlighted either the DSI (Fig 2A) or colitis scores (Fig 2B) and used the nearest neighbor search feature in GenePattern (HierarchicalClusteringViewer module) to generate Pearson (*r*) coefficients for all immune phenotypes relative to each disease endpoint, ranked from high (positive Pearson coefficient; directly correlated) to low (negative Pearson coefficient; inversely correlated). As expected, the absolute percentage of weight loss was the strongest direct correlate of the DSI (*r* = 0.865; *P* = 0.0026), the time post-T cell transfer was among the strongest inverse correlates of the DSI (*r* = -0.646; *P* = 0.0503), and histologic colitis did not correlate with the DSI (*r* = -0.045) (Fig 2A).

Interestingly, the percentage of induced Tregs (iTregs) in spleen was the strongest inversely correlated immune phenotype with weight loss (*r* = -0.758; *P* = 0.0179) (Fig 2B), suggesting splenic iTregs protect against weight loss in this model. As expected, iTreg frequency positively correlated with absolute iTreg numbers in spleen (Figure A in S1 File), and accordingly, absolute numbers of iTregs in spleen also inversely correlated with weight loss (*r* = -0.600; *P* = 0.087), albeit to a lesser degree than iTreg frequencies (Figure B in S1 File). By contrast, neither absolute numbers nor percentages of iTregs in other tissues (MLN, colon) correlated significantly with weight loss. Other immune phenotypes increased proportionately with weight loss, for example the percentage of ROR**γ**t^+^IL-22^+^ Teff cells in MLN (*r* = 0.859; *P* = 0.003), and the percentage of CXCR3^+^ Teff cells in colon (*r* = 0.7930; *P* = 0.011) (Fig 2B). Importantly, none of these correlates of weight loss showed similar relationships with the severity of histologic colitis (Fig 2B).

Whereas Pearson correlation is the default option for performing nearest neighbor searches in GenePattern, more common algorithms for assessing nearest neighbors calculate distance between variables (i.e., Euclidian distance, Manhattan distance) [12]. Nonetheless, the same immune phenotypes that correlated directly (e.g., ROR**γ**t^+^IL-22^+^ Teff cells in MLN, CXCR3^+^ Teff cells in colon) or inversely (iTregs in spleen) with T cell transfer-induced weight loss by Pearson coefficient were also identified as close or distant variables, respectively, by either Euclidian or Manhattan algorithms (Figure A-C in S2 File). Accordingly, these immune phenotypes also showed the strongest association with T cell transfer-induced weight loss using a cumulative rank order scoring system, which incorporates all 3 nearest neighbor algorithms (Figure D in S2 File).

A distinct set of immune phenotypes correlated with colitis severity, but not weight loss; the percentage of total Teff cells in colon increased proportionately with colitis severity (*r* = 0.714; *P* = 0.031), whereas frequency of both CCR6^+^ (*r* = -0.946; *P* = 0.0001) and **α**4**β**7^+^ (*r* = -0.642; *P* = 0.042) Teff cells in colon inversely correlated with colitis severity. Together, these results highlight the utility of using informatics in analyzing large immune profiling datasets, and support the notion that T cell transfer-induced weight loss and colitis are independent events driven by distinct immunologic mechanisms.

### Coupling immune phenotypes with clinical disease endpoints

**Fig 3:**
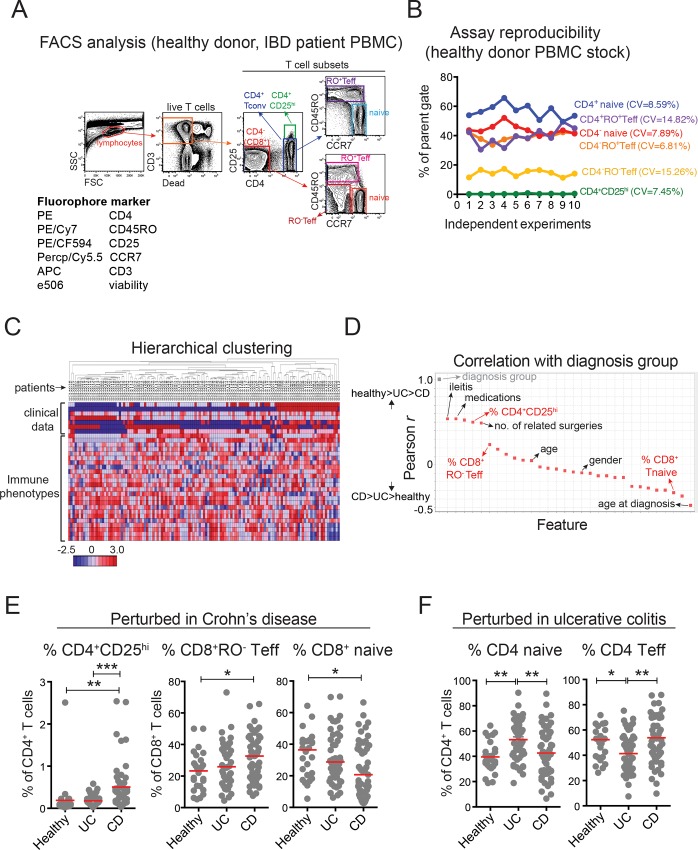
Informatics-based identification of immune dysregulation in clinical inflammatory bowel diseases. (A) *Bottom left*, 6-parameter FACS panel used for analyzing expression of surface antigens on peripheral blood mononuclear cells (PBMC) from healthy adult donors and adult IBD patients. Gating strategy for FACS analysis of human PBMC; immune subsets used in downstream analysis are indicated by gates and text. (B) Percentages of major T cell subsets in a healthy control PBMC stock, determined by repeated FACS analysis as in (A), over 10 independent staining experiments. Each subset is quantified based on the percentages within relevant parent gates (as in (A)); coefficients of variation (CVs) are indicated for each subset by color-matched text. (C) Heat map showing hierarchical clustering of 7 disease endpoints and 24 immune phenotypes in healthy adults (*n* = 26) and IBD patients ((ulcerative colitis (UC), *n* = 50; Crohn’s disease (CD), *n* = 53). (D) Rank-ordered (Pearson *r*) correlation values of all disease endpoints and immune phenotypes relative to diagnosis group (i.e., healthy donors, group 1; UC patients, group 2; CD patients, group 3). Relevant disease endpoints and immune phenotypes are indicated by black and red text, respectively; the correlation of the reference variable with itself (*r* = 1.0) is shown at top left in grey. (E) Immune cell subsets (CD4^+^CD25^hi^–*left*; CD8^+^RO^-^ Teff–*middle*; CD8^+^ naïve–*right*) identified by hierarchical clustering and ranked Pearson coefficients (as in (C, D)) perturbed in CD patient PBMC. (F) Immune cell subsets (CD4^+^ naive–*left*; CD4^+^ Teff–*right*) identified by hierarchical clustering and ranked Pearson coefficients (as in C and S3 File) perturbed in UC PBMC. Red lines indicate median values for each group. * P < .05, ** P < .01, *** P < .001, One-way ANOVA. Teff, effector/memory T cells. Only significant differences between groups are shown.

To validate this approach in a clinical setting, we performed FACS analysis on frozen PBMCs from healthy adult donors (*n* = 26), and adult IBD patients (ulcerative colitis (UC), *n* = 50; Crohn’s disease (CD), *n* = 53). For proof-of-principle, we assessed a relatively small number (*n* = 24) of manually gated immune parameters reflecting frequencies of major CD3^+^ and CD3^-^ lymphocyte subsets, including CD4^+^ and CD4^-^ (CD8^+^) CD3^+^ T cells; CD4^+^CD25^lo^ Tconv and CD4^+^CD25^hi^ cells (within CD3^+^CD4^+^ T cells); and CCR7^hi^CD45RO^-^ Tnaive, CCR7^lo^CD45RO^+^ Teff, and CCR7^lo^CD45RO^-^ Teff cells (within both CD4^+^ and CD8^+^ Tconv cells) (Fig 3A). To ensure assay reliability, we performed repeated analyses on a control stock of healthy donor PBMC, run in parallel during each independent experiment on sets of healthy donor and IBD patient samples. Coefficients of variation (CVs) for each major T cell subset ranged between 6–15% (Fig 3B), indicating reliable detection.

To enable hierarchical clustering and nearest neighbor searches with clinical and demographic data, we again converted disease and demographic parameters to single numeric values (Table 1). Clinical data included: diagnosis, age at diagnosis, disease activity (based on pathology), history of ileitis, number of related surgeries (e.g., bowel resection), and medication history. Demographic data included: age at collection, gender, and ethnicity (Table 2). All data were again collated for hierarchical clustering (Fig 3C).

**Table 2:** Demographic data of healthy volunteers and IBD patients analyzed in the study.

	Healthy controls	Ulcerative colitis	Crohn’s disease
Number	26	50	53
**Age**			
range	23–67	22–72	21–88
mean ± SD	36±14	37±15	40±16
**Gender**			
male	10	29	28
female	16	21	25
**Ethnicity**			
Caucasian	3	35	37
Hispanic	0	0	2
African	1	0	2
Asian	2	2	3
Other minority	1	10	5
Unknown	21	3	3

Number, age, gender and ethnicities of healthy adult volunteers (healthy controls) or inflammatory bowel disease (IBD) patients–ulcerative colitis, Crohn’s disease–profiled in this study.

We performed two nearest neighbor searches to extract immune correlates of CD (Fig 3D) or UC (S3 File). As expected, clinical parameters such as ileitis (*r* = 0.565; *P* < 0.0001), and medications (*r* = 0.514; *P* < 0.0001) directly correlated with CD, whereas age at diagnosis was the strongest inverse correlate of both CD and UC (Fig 3D and S3 File). More importantly, our analyses identified distinct nodes of T cell dysregulation in CD and UC. In CD, percentages of both CD4^+^CD25^hi^ cells and CD8^+^RO^-^ Teff cells were increased, and percentages of CD8^+^ naive cells were decreased relative to both healthy controls and UC patients (Fig 3E). By contrast, UC patients displayed increased percentages of CD4^+^ naive cells, and decreased percentages of CD4^+^RO^+^ Teff cells *vs*. either healthy donors or CD patients (Fig 3F).

## Discussion

Here we describe a simple informatics pipeline that can be leveraged to rapidly discriminate immune correlates of clinical and pre-clinical disease parameters within high-content cytometry datasets. In mice, we show that distinct immune profiles correlate with T cell transfer-induced weight loss or histologic colitis. These results are interesting because they suggest that weight loss and colitis in this model are unrelated; they are important because these endpoints are widely used in the laboratory and presented in the literature as interchangeable measures of disease [13,17]. In the colon lamina propria, for example, we show that CXCR3^+^ Teff cells increase proportionately with more severe weight loss, but show no correlation with severity of mucosal inflammation. By contrast, CCR6^+^ Teff cells decrease commensurate with more severe histologic inflammation but show no association with weight loss. Expression of CXCR3 in Teff cells enriches for IFN**γ**-producing Th1 cells, whereas CCR6 expression broadly distinguishes ROR**γ**t^+^ Th17 cells that transiently express IL-17A and IL-17F pursuant to signals present in the local microenvironment [18–20]. The strong inverse correlation between CCR6^+^ Th17 cell abundance and histologic colitis (*r* = -0.946) points to a protective function of CCR6^+^ Th17 cells in the colonic mucosa, which is both consistent with the fact that Th17 cytokines enforce barrier function in the gut [16] and supported by previous data from both animal models of IBD and clinical trials; Th17 cells are capable of suppressing experimental colitis in mice [21], and the neutralizing IL-17A antibody, Secukinumab, not only failed to show efficacy in a recent IBD clinical trial, it exacerbated disease activity [22].

The association between mucosal CXCR3^+^ Th1 cells and T cell transfer-induced weight loss is perhaps more difficult to understand given that the mechanisms underlying weight loss in this model are ill defined. Th1 cell-derived IFN**γ** is known classically for activating phagocytes and cytolytic lymphocytes (e.g., natural killer [NK] cells), and has been experimentally shown to promote parenchymal cell death and tissue damage in a variety of autoimmune mouse models [21,23,24]. Thus, it is possible that Th1 cells induce histologically subtle damage to the colonic epithelium, leading in turn to dissemination of bacterial products from the gut (e.g., to the liver) and failure to thrive [25]. Indeed, additional methods to assess intestinal pathology, such as 3-dimensional stereomicroscopy, could be useful to distinguish qualitatively distinct mucosal lesions [26]. It is also possible that increased frequency of CXCR3^+^ Th1 cells in colons of morbid animals is a consequence–rather than cause–of excessive tissue damage. Expression of IFN**γ** *per se* showed less strong correlations than CXCR3 with T cell transfer-induced weight loss (data not shown), and CXCR3 ligands, such as CXCL9, CXCL10, CXCL11, are broadly expressed by endothelial and epithelial cells, where they are upregulated upon microbial infection, stress, or tissue damage [27].

Frequencies of colonic **α**4**β**7 (integrin)^+^ Teff cells also inversely correlated with severity of histologic colitis. This result, which implies a protective role of **α**4**β**7^+^ Teff cells in this model, is surprising given that **α**4**β**7 is functionally required for experimental colitis in mice [28] and the neutralizing **α**4**β**7 antibody, Vedolizumab, is now approved for use as a therapeutic in IBD [29,30]. Like most integrins, however, cell surface **α**4**β**7 is labile and can be internalized upon T cell activation [31], and we consistently observed lower **α**4**β**7 staining in cells from colon lamina propria *vs*. either spleen or MLN (data not shown). Thus, the apparent increase in Teff cell **α**4**β**7 expression in less inflamed colons may simply reflect reduced T cell activation in response to luminal antigens, which is also required for colitis in this model [32]. As a whole, these results highlight how informatics-based analyses of flow cytometry data can be used, both to gain insight into disease biology and identify biomarkers linked to discrete disease endpoints.

In humans, we identified non-overlapping nodes of immune dysregulation in the two common forms of IBD, CD and UC. Despite their common classification, CD and UC are distinct diseases that affect discrete regions of the intestinal tract, present as different histopathologic lesions, and display unique genetic susceptibilities [33,34]. It is interesting in this regard that CD was associated with perturbations mostly in CD8^+^ T cell subsets, whereas alterations in the CD4^+^ T cell compartment were most obvious in UC. The specific dysregulation of CD8^+^ T cells in CD patient blood observed here is consistent with at least two previous reports, including one from van Unen et al., which used a panel of 32 metal isotope-tagged antibodies and CyTOF to profile nearly 150 immune cell phenotypes in blood and mucosal biopsies of CD patients [8,35]. This shift from naïve (CCR7^hi^RO^-^) to chronically activated (CCR7^-^RO^-^) CD8^+^ T cells in the blood of CD patients predicts the previously reported infiltration of activated CD8^+^ T cells into small bowel lesions of both ileitis-prone mice and human CD patients [36,37]. We are not aware of other reports documenting similar changes in naïve and effector/memory CD4^+^ T cells in UC patient peripheral blood. The extent to which these immune profiles can be generalized within CD and UC patients requires further investigation, but these preliminary results highlight the unique biology at play in CD and UC, and predict the non-overlapping clinical responses of CD and UC patients to targeted therapies [29,30,38].

Technically, there are 3 major advantages offered by an informatics view of cytometry data. First is expedience; analyzing dozens to hundreds of distinct parameters in groups of mice or humans “one-by-one” takes days to weeks. By contrast, this same analysis performed via informatics takes minutes. Second is breadth; cluster-based analysis affords a comprehensive view of all relationships between immune phenotypes and clinical or pre-clinical disease endpoints. Indeed, discriminating immune features that are not associated with disease can be equally as informative as understanding those that are. Third is sensitivity; we show several examples where subtle changes in immune cell frequencies correlate significantly with disease endpoints. For example, the percentage of CD4^+^ iTreg cells in spleen strongly correlated with protection from T cell transfer-induced weight loss (*r* = -0.758; *P* = 0.0179) (Fig 2B, *top left*), despite the fact that iTregs represented only 0.45–2.66% of total splenic CD4^+^ T cells in this cohort of animals. This information could have been lost if not for unbiased informatics analyses.

The notion of using informatics to handle ultra-high content cytometry data is in itself, not new, and several tools have been developed in recent years, both to enable unsupervised gating of raw cytometry data (e.g., Citrus, AutoGate) [4,5], and to visualize subset abundance and lineage relationships within patient populations (e.g., SPADE) [6]. Yet whether using manual or data-driven methods of raw cytometry data analysis, the question of how one relates these phenotypes to myriad clinical or pre-clinical disease parameters has remained a challenge. Hierarchical clustering of manually analyzed cytometry data has also been used previously to display qualitative differences between cell types or healthy *vs*. diseased patients [39,40]. Our study now shows added value of performing downstream nearest neighbor searches to identify quantitative relationships between immune phenotypes and select clinical or pre-clinical variables. Most importantly, this analysis platform uses publically available software, can be retrospectively applied to existing datasets, and is suitable for any clinical or pre-clinical disease setting.

## Supporting Information

S1 File Relationship between iTreg frequency, absolute number and T cell-induced weight loss. (A) Correlation between frequencies (i.e., percentage of total CD4^+^ T cells) and absolute numbers of CD25^+^Foxp3^+^ induced T regulatory cells (iTregs) in spleens of colitic FVB.*Rag1*^-/-^ mice transplanted with wild type naïve CD4^+^ T cells (as in Fig 1A). iTreg frequencies were determined by gating in FlowJo following *ex vivo* intracellular FACS analysis (as in Fig 1D). iTreg numbers were calculated by multiplying the total number of mononuclear cells recovered from spleen by subset frequencies (e.g., percentage of parent gates; example shown in Fig 1D). (B) Correlation between iTreg numbers in spleen and T cell transfer-induced weight loss (disease severity). Pearson (*r*) coefficients are indicated in red text; ** P < .01, Pearson correlation test.
(TIF)

S2 File Relationship between GenePattern nearest neighbor search algorithms. Correlation between Pearson (*r*) coefficients (correlation with T cell transfer-induced weight loss) and Euclidian (A) or Manhattan (B) distances (distance from T cell transfer-induced weight loss) of immune phenotypes in colitic FVB.*Rag1*^-/-^ mice. (C) Correlation between Euclidian and Manhattan distances (from T cell transfer-induced weight loss) of immune phenotypes in colitic FVB.*Rag1*^-/-^ mice. Pearson (*r*) coefficients are indicated in red text; immune phenotypes identified by Pearson coefficients (in Fig 2A and 2C) are highlighted by blue text and arrowheads. **** P < .0001, Pearson correlation test. (D) Combined rank order score of all pre-clinical and immunophenotypic variables relative to T cell transfer-induced weight loss following nearest neighbor searches using Pearson coefficient, Euclidian distance, and Manhattan distance. For Pearson correlation, variables were sorted from low (inverse) to high (direct) Pearson (*r*) coefficients and given low-to-high rank order scores. For Euclidian and Manhattan distance searches, variables were sorted from high-to-low dissimilarity values and given low-to-high rank order scores. The combined rank order score reflects the sum of all 3 rank order values; variables with highest combined rank order scores are increased in T cell-transferred FVB.*Rag1*^-/-^ mice showing the greatest weight loss. Immune phenotypes identified by Pearson coefficients (in Fig 2A and 2C) are highlighted in blue.
(TIF)

S3 File Immunophenotypic correlates of ulcerative colitis. Rank-ordered (Pearson *r*) correlation values of all disease endpoints and immune phenotypes relative to diagnosis group (i.e., healthy donors, group 1; CD patients, group 2; UC patients, group 3). Relevant disease endpoints and immune phenotypes are indicated by black and red text, respectively; the correlation of the reference variable with itself (*r* = 1.0) is shown at top left in grey.
(TIF)
