# Supplementary figures and images for: Informatics-Based Discovery of Disease-Associated Immune Profiles
Source: PLoS One. 2016 Sep 26;11(9):e0163305. doi: 10.1371/journal.pone.0163305 (PMC5036861; doi:10.1371/journal.pone.0163305)

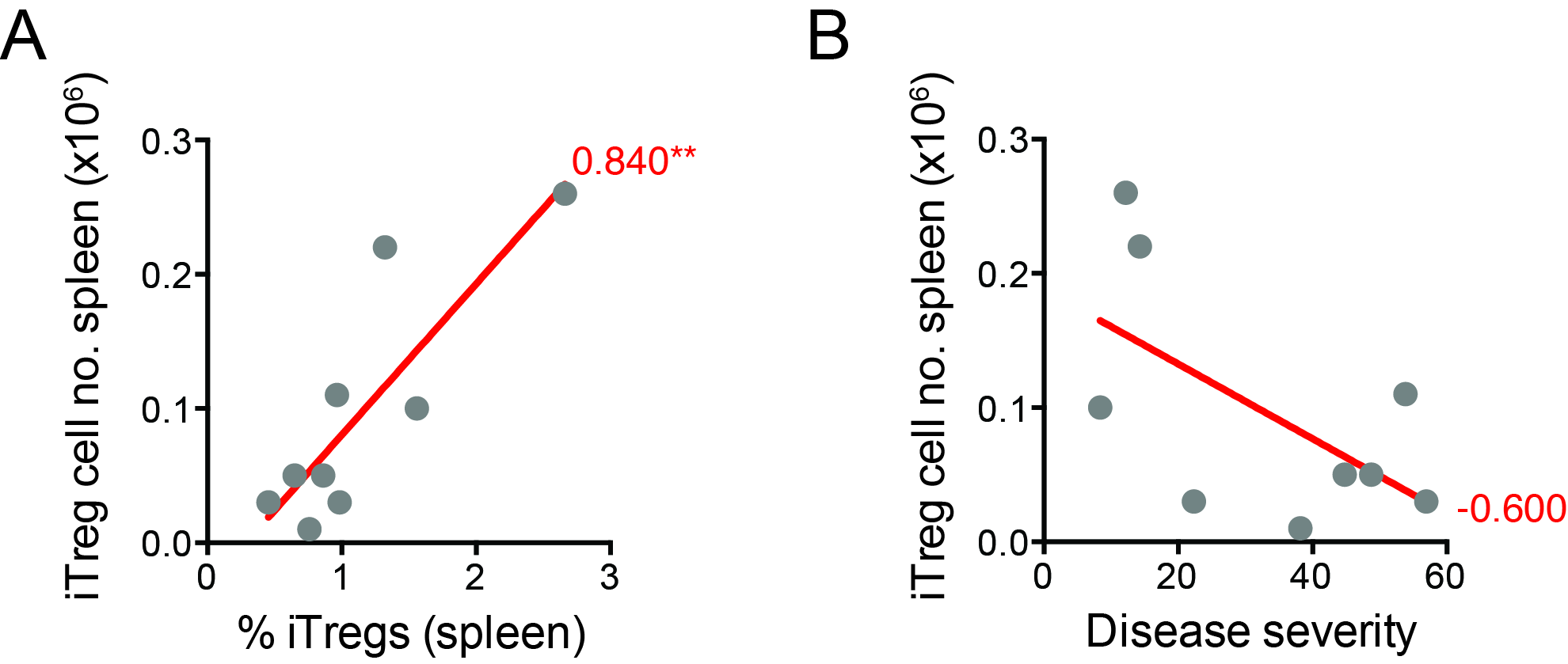

Supplement: S1 File — (A) Correlation between frequencies (i.e., percentage of total CD4+ T cells) and absolute numbers of CD25+Foxp3+ induced T regulatory cells (iTregs) in spleens of colitic FVB.Rag1-/- mice transplanted with wild type naïve CD4+ T cells (as in Fig 1A). iTreg frequencies were determined by gating in FlowJo following ex vivo intracellular FACS analysis (as in Fig 1D). iTreg numbers were calculated by multiplying the total number of mononuclear cells recovered from spleen by subset frequencies (e.g., percentage of parent gates; example shown in Fig 1D). (B) Correlation between iTreg numbers in spleen and T cell transfer-induced weight loss (disease severity). Pearson (r) coefficients are indicated in red text; ** P < .01, Pearson correlation test. (TIF) [file pone.0163305.s001.tif]

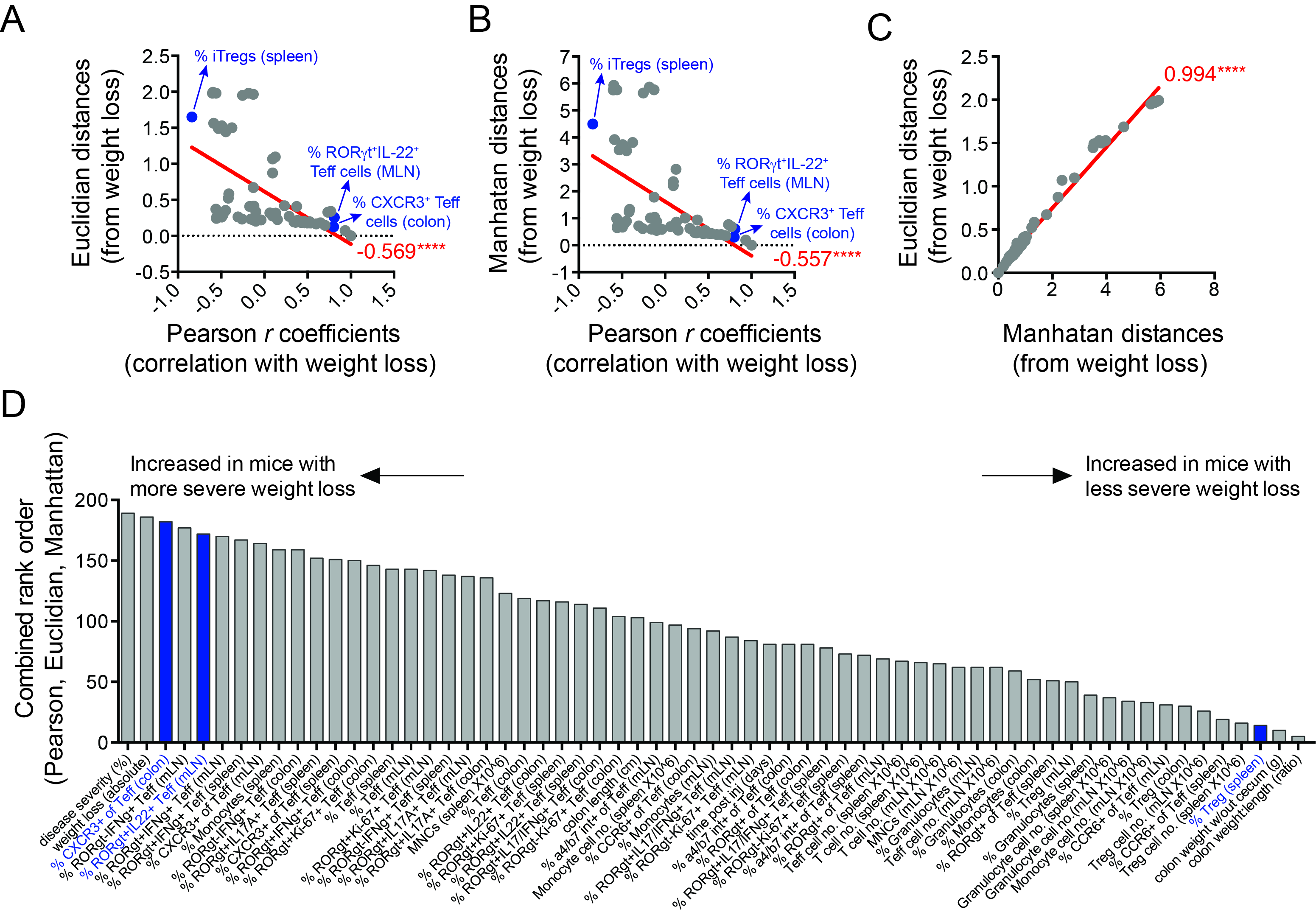

Supplement: S2 File — Correlation between Pearson (r) coefficients (correlation with T cell transfer-induced weight loss) and Euclidian (A) or Manhattan (B) distances (distance from T cell transfer-induced weight loss) of immune phenotypes in colitic FVB.Rag1-/- mice. (C) Correlation between Euclidian and Manhattan distances (from T cell transfer-induced weight loss) of immune phenotypes in colitic FVB.Rag1-/- mice. Pearson (r) coefficients are indicated in red text; immune phenotypes identified by Pearson coefficients (in Fig 2A and 2C) are highlighted by blue text and arrowheads. **** P < .0001, Pearson correlation test. (D) Combined rank order score of all pre-clinical and immunophenotypic variables relative to T cell transfer-induced weight loss following nearest neighbor searches using Pearson coefficient, Euclidian distance, and Manhattan distance. For Pearson correlation, variables were sorted from low (inverse) to high (direct) Pearson (r) coefficients and given low-to-high rank order scores. For Euclidian and Manhattan distance searches, variables were sorted from high-to-low dissimilarity values and given low-to-high rank order scores. The combined rank order score reflects the sum of all 3 rank order values; variables with highest combined rank order scores are increased in T cell-transferred FVB.Rag1-/- mice showing the greatest weight loss. Immune phenotypes identified by Pearson coefficients (in Fig 2A and 2C) are highlighted in blue. (TIF) [file pone.0163305.s002.tif]

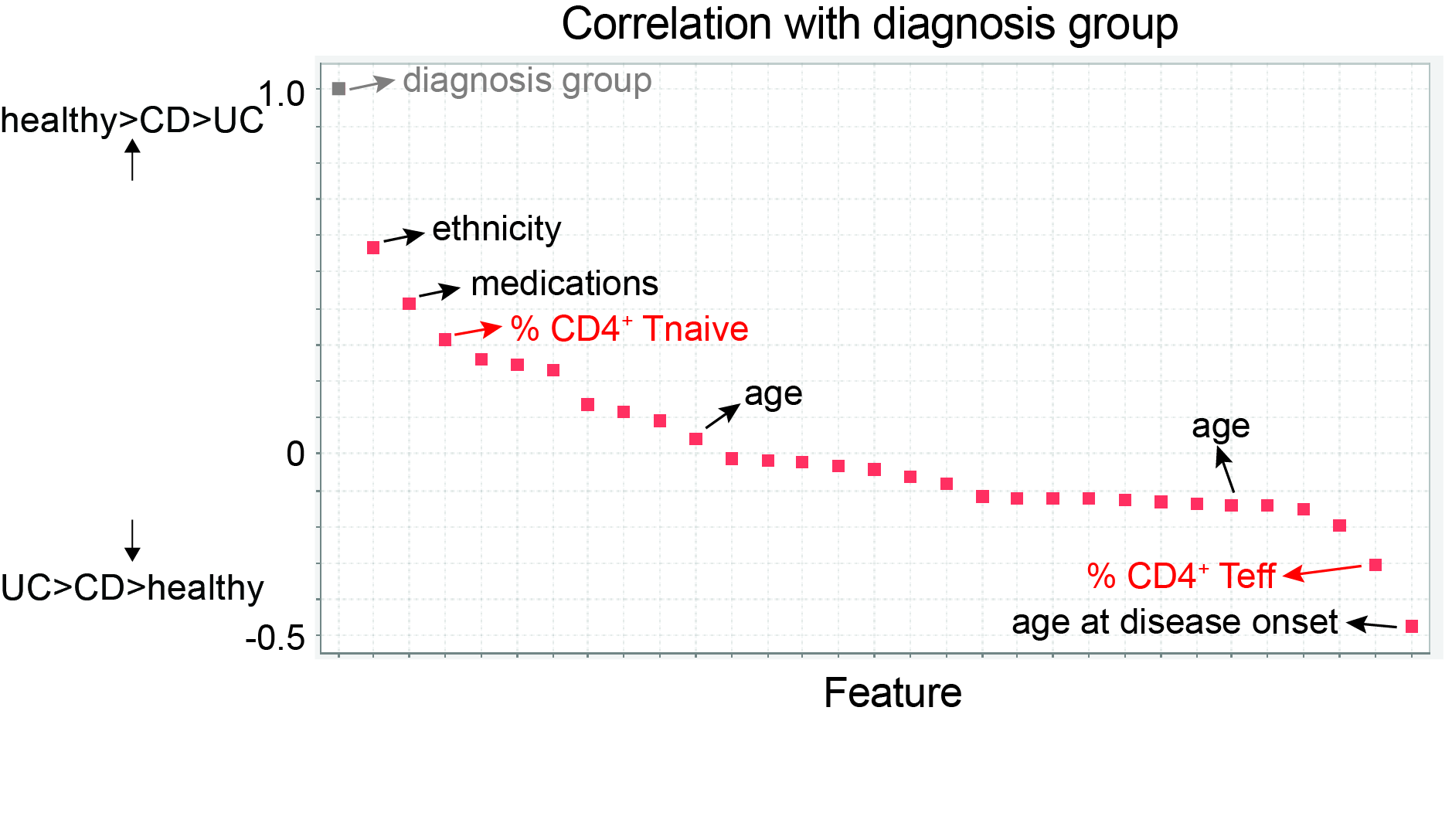

Supplement: S3 File — Rank-ordered (Pearson r) correlation values of all disease endpoints and immune phenotypes relative to diagnosis group (i.e., healthy donors, group 1; CD patients, group 2; UC patients, group 3). Relevant disease endpoints and immune phenotypes are indicated by black and red text, respectively; the correlation of the reference variable with itself (r = 1.0) is shown at top left in grey. (TIF) [file pone.0163305.s003.tif]
